# Evolution of *Streptococcus pneumoniae* and Its Close Commensal Relatives

**DOI:** 10.1371/journal.pone.0002683

**Published:** 2008-07-16

**Authors:** Mogens Kilian, Knud Poulsen, Trinelise Blomqvist, Leiv S. Håvarstein, Malene Bek-Thomsen, Hervé Tettelin, Uffe B. S. Sørensen

**Affiliations:** 1 Institute of Medical Microbiology and Immunology, Aarhus University, Aarhus, Denmark; 2 Department of Chemistry, Biotechnology and Food Science, Norwegian University of Life Sciences, Ås, Norway; 3 Institute for Genome Sciences, Department of Microbiology and Immunology, University of Maryland School of Medicine, Baltimore, Maryland, United States of America; Centre for DNA Fingerprinting and Diagnostics, India

## Abstract

*Streptococcus pneumoniae* is a member of the Mitis group of streptococci which, according to 16S rRNA-sequence based phylogenetic reconstruction, includes 12 species. While other species of this group are considered prototypes of commensal bacteria, *S. pneumoniae* is among the most frequent microbial killers worldwide. Population genetic analysis of 118 strains, supported by demonstration of a distinct cell wall carbohydrate structure and competence pheromone sequence signature, shows that *S. pneumoniae* is one of several hundred evolutionary lineages forming a cluster separate from *Streptococcus oralis* and *Streptococcus infantis*. The remaining lineages of this distinct cluster are commensals previously collectively referred to as *Streptococcus mitis* and each represent separate species by traditional taxonomic standard. Virulence genes including the operon for capsule polysaccharide synthesis and genes encoding IgA1 protease, pneumolysin, and autolysin were randomly distributed among *S. mitis* lineages. Estimates of the evolutionary age of the lineages, the identical location of remnants of virulence genes in the genomes of commensal strains, the pattern of genome reductions, and the proportion of unique genes and their origin support the model that the entire cluster of *S. pneumoniae*, *S. pseudopneumoniae*, and *S. mitis* lineages evolved from pneumococcus-like bacteria presumably pathogenic to the common immediate ancestor of hominoids. During their adaptation to a commensal life style, most of the lineages gradually lost the majority of genes determining virulence and became genetically distinct due to sexual isolation in their respective hosts.

## Introduction

Phylogenetic analysis based on 16S rRNA sequences of type strains of the more than 50 *Streptococcus* species reveal a clustering pattern that reflect their pathogenic potential and ecological preferences [1], [2]. One exception is the Mitis group, which contains one of the leading pathogens affecting mankind, *Streptococcus pneumoniae*, along with 11 species that are prototype commensals of the upper respiratory tract. *S. pneumoniae* is strikingly similar to the three commensal species *Streptococcus mitis*, *Streptococcus oralis*, and *Streptococcus infantis* often causing problems of identification in clinical microbiology laboratories [3]–[5]. Introduction of the species *Streptococcus pseudopneumoniae* [6] emphasized this problem. Properties that explain the pathogenic potential of *S. pneumoniae* include a polysaccharide capsule, IgA1 protease, pneumolysin, autolysin, and several surface-exposed proteins that mediate contact with components of host tissues and secretions [7]–[9].

Most members of the Mitis group of streptococci are naturally competent for genetic transformation and produce well-characterized competence pheromones (CSPs) and pheromone receptors [10], [11]. Accordingly, homologous recombination is believed to play a major role in the evolution of these bacteria, which is reflected in mosaic structures in gene sequences [12]–[15], and may have important implications for the circumscription of individual species [16], [17]. Expression by occasional isolates of the commensal species of proteins that are known to contribute to virulence in *S. pneumoniae* has been taken as evidence of horizontal gene transfer [3], [18]–[20]. Such data led Hakenbeck and coworkers [18] to suggest “a smooth transition” between these species. To what extend transitional forms are involved in occasionally reported cases of meningitis, septicemia, toxic-shock-like syndrome, and eye infections attributed to the otherwise commensal species is not known.

The aim of this study was to investigate the evolutionary history of the pneumococcus and its close commensal relatives using a polyphasic phylogenetic strategy. By analysis of this unique cluster of closely related species with very distinct pathogenic potentials we were able to propose a model for the evolution of pathogenic and commensal streptococci. We further show that related commensal streptococci show genetic diversification to an extent that challenges current definitions of prokaryotic species.

## Results

### Phylogenetic Reconstruction

Alignment of the sequences of the four housekeeping genes, *ddl*, *gdh*, *rpoB*, and *sodA*, obtained for 118 strains tentatively assigned to *S. pneumoniae*, *S. mitis*, *S. oralis*, and *S. infantis* plus eight sets of sequences extracted from *S. pneumoniae* genomes revealed a remarkable sequence polymorphism resulting in a total of 92 *ddl* alleles (mean distance 0.105±0.011), 99 *gdh* alleles (mean distance 0.111±0.009), 95 *rpoB* alleles (mean distance 0.045±0.005), and 94 *sodA* alleles (mean distance 0.053±0.006). Virtually all shared alleles were found among strains of *S. pneumoniae*. Identical sequences in all loci were found in some reference strains assigned to *S. mitis* (NCTC 8029/SK24, NCTC 8031/SK320, and ATCC 12398/SK319) and *S. infantis* (CCUG 25812 and CCUG 25857). Although listed as separate strains in the respective strain collection catalogues, they originate in the same laboratory and we assume that they represent the same isolate (NCTC 8031 is erroneously listed as *Lactococcus lactis subsp. lactis* in the NCTC cataloque). As a consequence, shared *ddl*, *gdh*, *rpoB*, or *sodA* alleles among strains assigned to *S. mitis*, *S. oralis*, or *S. infantis* were virtually non-existent. The sequences reported in this paper have been deposited in GenBank (http://www.ncbi.nlm.hih.gov/Genbank) under accession numbers EU797799-798270.

**Figure 1:**
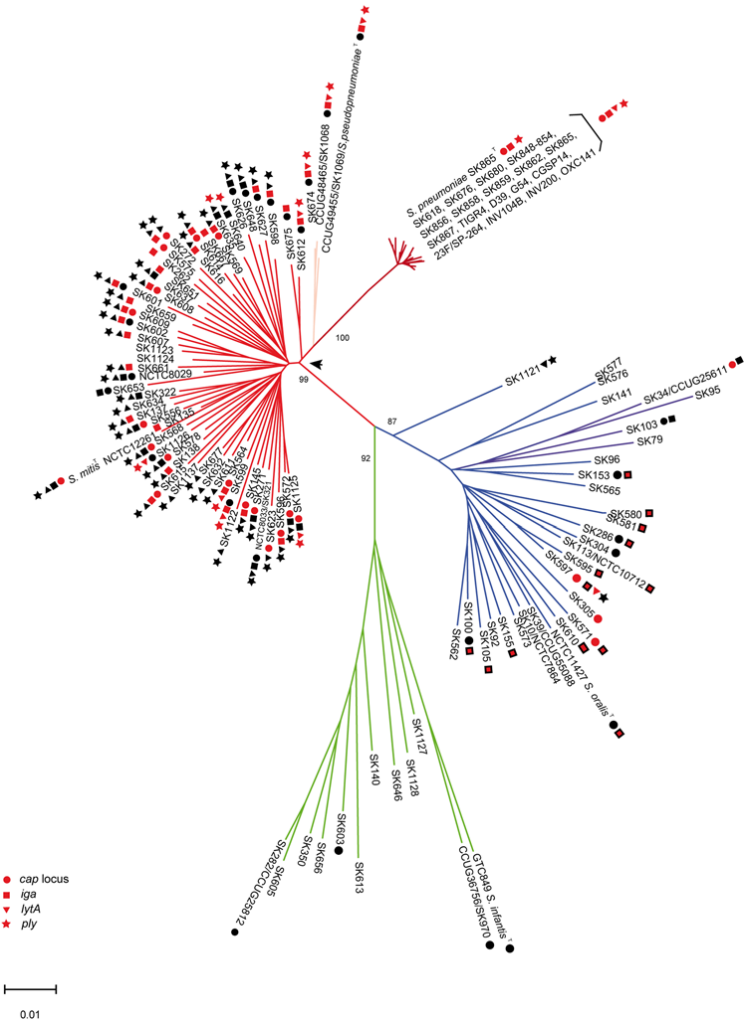
Phylogenetic tree constructed with the minimum evolution algorithm in MEGA version 4.0 and based on concatenated partial sequences of the house-keeping enzyme genes *ddl*, *gdh*, *rpoB*, and *sodA*. Type strains of individual species are shown with species designation. Bootstrap values (%) are based on 1000 replications. The three major clusters supported by significant bootstrap values are the pneumoniae-mitis-pseudopneumoniae cluster (red lines), the Oralis cluster (blue lines), and the Infantis cluster (green lines). The subcluster of *S. pneumoniae* strains within the pneumoniae-mitis-pseudopneumoniae cluster is indicated by dark red lines (ruby), *S. pseudoneumoniae* strains within the pneumoniae-mitis-pseudopneumoniae cluster are indicated by pink, and strains previously assigned to “*S. mitis* biovar 2” within the Oralis cluster are indicated by dark blue lines. The random presence of homologues of virulence factors usually associated with *S. pneumoniae* (*cap* locus, capsule synthesis operon; *iga*, IgA1 protease gene; *lytA*, autolysin gene; *ply*, pneumolysin gene) in the diverse population of Mitis lineages is illustrated. A red signature indicates presence of the virulence gene, and black signature indicates a PCR product size compatible with absence of the gene. Black squares with a red center indicate IgA1 protease activity but an amplicon size in support of lack of an *iga* gene in the context found in *S. pneumoniae*. No signature indicates lack of a PCR product presumably due to no match of the primers. The arrow indicates the hypothetical immediate common ancestor of the red cluster. The scale bar refers to genetic divergence as calculated by the MEGA software.

A phylogenetic tree based on concatenated sequences (1716 bp) of *ddl-gdh-rpoB-sodA* showed three major monophyletic clusters supported by bootstrap values of 87 to 99 (Figure 1). One of the three clusters containing more than 50 deeply branching lineages included type strains of *S. pneumoniae*, *S. pseudopneumoniae*, and *S. mitis* (red cluster in Figure 1). Within this cluster the lineage containing the *S. pneumoniae* type strain branched off into a tight sub-cluster of 25 strains including eight strains represented by sequences extracted from *S. pneumoniae* genomes. With the exception of the lineage containing the *S. pseudopneumoniae* type strain and two other relatively close strains, virtually all of the remaining lineages consisted of only a single strain. We cannot exclude that the lack of branching of the *S. mitis* lineages compared to that of the *S. pneumoniae* lineage is a result of overrepresentation of disease-associated strains compared to the individual lineages of commensal non-pneumococcus isolates. We refer to the entire red cluster of 84 strains as the pneumoniae-mitis-pseudopneumoniae cluster. The second cluster (blue cluster in Figure 1), likewise, comprised numerous deeply branching lineages one of which included the type strain of *S. oralis*. One of the subclusters (dark blue) of this cluster were composed of four strains previously referred to as “*S. mitis* biovar 2” [21], which clearly is more related to *S. oralis* than to *S. mitis* based on these data. The third very diffuse cluster (green cluster in Figure 1) included the type strain of *S. infantis* and several other reference collection strains received under that designation.

To further evaluate the clustering we analyzed the cell wall polysaccharide structure of all strains. Two monoclonal antibodies were used to detect epitopes characteristic of the backbone and the phosphocholine residues of C-polysaccharide, respectively. We have previously demonstrated that reactivity with both antibodies is indicative of the phosphocholine-containing C polysaccharide, which constitutes the Lancefield group O antigen [22]. This structure was present in all 17 *S. pneumoniae* strains and, with 5 exceptions, in all other 57 strains of the pneumoniae-mitis-pseudopneumoniae cluster. Two of the 5 exceptional strains (SK598 and SK601) lacked the phosphocholine residue of the C-polysaccharide/group O antigen. We previously showed that ethanolamine substitutes for phosphocholine in such strains [23]. The remaining three strains showed evidence of phosphocholine but lacked the characteristic epitope of the backbone of C polysaccharide. While phosphocholine was a component also of the cell wall polysaccharide of all 28 strains of the Oralis cluster in agreement with previous observations [24], they all lacked the backbone characteristic of the C-polysaccharide/group O antigen. These observations provide further support for the two major clusters in Figure 1. Strains of the deeply branching “Infantis cluster” showed variable presence of the two epitopes.

Sequence comparison of 16S rRNA genes has become a standard method for phylogenetic analysis [25]. Cluster analysis of a stretch of 439 to 449 nucleotides corresponding to positions 51 to 493 in the *Escherichia coli* 16S rRNA gene, which is the phylogenetically informative part of the 16S rRNA gene in streptococci, confirmed that the strains in Figure 1 are unrelated to the remaining species of Mitis group streptococci. However, analysis of 73 representatives of the strains included in Figure 1 failed to reveal clusters supported by significant bootstrap values (Figure S1). In agreement with previous observations [26] this is, in part, due to conservation of the 16S rRNA sequence within the Mitis group of streptococci.

The genomes of the streptococci of this study harbor four copies of the rRNA operon. In four strains from the *S. oralis* cluster the initial 16S rRNA sequences were ambiguous. Sequencing of individual 16S rRNA genes in these strains revealed two distinct alleles closely similar to the *S. oralis* type strain and the *S. mitis* type strain, respectively (Figure S2). This intragenomic polymorphism, similar to that reported for occasional strains of other bacterial species [27], is direct evidence of homologous recombination between the two major clusters in Figure 1. The unequivocal affiliation of these four strains with rRNA gene polymorphisms with *S. oralis* in the *ddl-gdh-rpoB-sodA* tree (Figure 1), was supported by DNA-DNA hybridization (71.0% and 70.3% homology of SK100 and SK105 to the *S. oralis* type and 54.8% and 52.8% homology to the *S. mitis* type, respectively). Combined these data strongly suggest transfer of gene sequences from *S. mitis* to *S. oralis*. Theoretically, the presence of two pairs of identical sequences would either require two independent events of homologous recombination affecting two of the four gene copies or an aborted intragenomic repair/homogenization as a divergent copy resulting from a homologous recombination event is assumed to be corrected by a rapid intragenomic homogenization process [28]. Combined with equivocal clustering based on 16S rRNA sequences, these findings invalidate the use of 16S rRNA sequences for species differentiation in this part of the Mitis group of *Streptococcus*.

### Extensive ComC Polymorphism

To obtain further comparative data on the genetic relationships and divergence within the clusters we determined the sequence of the competence-inducing peptide ComC of 66 selected strains. The amino acid sequences showed a remarkable diversity (Table S2). Two major pheromone types (pherotypes) have been described within natural populations of *S. pneumoniae* and a few additional pherotypes have been reported for occasional isolates [for review see ref. 33]. Remarkably, with few exceptions, each of the strains of the pneumoniae-mitis-pseudopneumoniae cluster, excluding *S. pneumoniae*, possessed a unique ComC sequence. Numerous distinct ComC sequences and pherotypes were also detected among strains of the *S. oralis* and *S. infantis* clusters. In spite of this significant polymorphism, a distinct signature sequence in the ComC leader peptide was observed for each of the three phylogenetic clusters (Table 1 and Table S1). It is assumed that the ComC leader sequence is recognized by the dedicated CSP transporter ComA, conceivably explaining why the leader is relatively conserved. Interestingly, one *S. mitis* strain (SK145) had a ComC leader sequence representing a chimera between typical *S. mitis* and *S. oralis* signatures, and one *S. pneumoniae* strain (SK676) had a ComC with a leader sequence signature identical to that of *S. oralis* strains (Table S1). Both of these aberrant sequences suggest a recombination event affecting the *comC* gene. Switching of CSP among strains of streptococci was previously reported [34]. Overall, these findings further support the clustering based on housekeeping gene sequences and underscore the significant genetic polymorphisms within the clusters.

**Table 1:** Cluster-specific amino acid sequence signatures in the leader of ComC.

Species	Amino acid sequence signatures
*S. pneumoniae*	----------v--k—d--------**-----------------········**
*S. mitis*	
*S. pseudopneumoniae*	
*S. oralis*	----------k--t--e-------**------------------···**
*S. infantis*	-------------q--n--e----**-----------------···**

Amino acid residues of mature CSP are indicated by bold lines. Dots illustrate the number of amino acids by which respective sequences of the mature CSP may vary in length.

### Evidence of Limited Homologous Recombination between Clusters

The alignments of housekeeping gene sequences showed clear mosaic structures suggestive of homologous recombination having contributed to the long-term evolution of the observed alleles. To examine if interspecies recombination contributes to this process we compared phylogenetic patterns deduced from each of the four sequenced housekeeping genes. For all 124 strains, save four, the clustering based on each of the four gene sequences was concordant. The most striking exception was one strain (SK597), which according to *ddl*, ComC sequences, and cell wall polysaccharide type was part of the *S. mitis* complex but in trees based on *gdh*, *rpoB*, and *sodA* sequences clustered with *S. oralis*. The remaining three strains were all part of the Infantis cluster and each had one or two loci (*ddl* and/or *sodA*) that were reminiscent of *S. mitis* or *S. oralis*. Interestingly, the tree strains in the lineage that included the *S. pseudopneumoniae* type had a *sodA* allele typical of *S. pneumoniae* whereas all other loci were typical of *S. mitis*.

To further explore the extent of allelic exchange between recognized species we analyzed an overlapping 460 base pair (bp) stretch of our 124 *gdh* sequences and the 146 *gdh* alleles that have been detected among 2612 sequence types of *S. pneumoniae* (www.mlst.net). Seven of the *S. pneumoniae gdh* alleles (*gdh* alleles 20, 40, 57, 94, 97, 107, and 117, represented in a total of 23 sequence types) clustered within the pneumoniae-mitis-pseudopneumoniae cluster outside the *S. pneumoniae* lineage. None clustered with the Oralis and Infantis clusters.

Combined with the described intragenomic 16S rRNA gene polymorphism and the mentioned few aberrant and chimeric ComC amino acid sequences, the occasional in-congruent phylogenetic topologies for individual loci are indicative of very limited homologous recombination between clusters. The different frequencies of recombination affecting different loci and different pairs of species are likely to reflect the extent of sequence divergence [35], which conceivably has become a barrier to efficient recombination between the Mitis and Oralis clusters with the exception of loci that contain highly conserved sequences such as the 16S rRNA gene. Using an identical strategy we previously demonstrated a significantly higher prevalence of recombination affecting clinical isolates of *S. sanguinis* (27%) and streptococci belonging to the Anginosus group (14%)[36].

### Genetic Distances, DNA Homology and Taxonomic Inference

The within-group genetic distance for the 25 strains of the *S. pneumoniae* cluster based on concatenated *ddl-gdh-rpoB-sodA* sequences was calculated to be 0.010±0.003 (Mean±standard error of the mean) closely similar to the value calculated on the basis of concatenated sequences of six loci (*aroE*, *gdh*, *gki*, *recP*, *spi*, *xpt*) in the 2612 recognized sequence types of *S. pneumoniae* (www.mlst.net), i.e., 0.010±0.001, and similar to the distance that may be calculated for several other species of pathogenic bacteria for which comprehensive sequence data are available (not shown). In striking contrast, the corresponding values were 0.086±0.007 for the remaining part of the pneumoniae-mitis-pseudopneumoniae cluster, and 0.097±0.008 for the Oralis cluster. To evaluate to what extent the extensive sequence diversity among strains of the Mitis lineages was reflected in the overall level of DNA homology between strains we compared seven selected strains to the type strain of *S. mitis* (NCTC 12261). By tradition, DNA homology of more than 70% determined in a DNA-DNA hybridization assay is expected for two strains belonging to the same species [37]. In concordance with the significant sequence divergence only one of seven stains (SK138) of the Mitis lineages showed a hybridization value exceeding 70% (70.2%). The remaining strains showed hybridization values of 49.2 (SK612), 50.9 (SK667), 59.6 (SK596), 59.6 (SK319), 60.3 (NCTC 8029/SK24), and 67.1% (SK137) to the *S. mitis* type strain. These values indicate that the red cluster in Figure 1, which contains the type strains of the recognized species *S. pneumoniae*, *S. pseudopneumoniae*, and *S. mitis*, consists of a considerable number of separate evolutionary lineages most of which represent distinct species according to current taxonomic standards. No direct correlation between DNA-DNA hybridization values and sequence divergence in the four sequenced loci could be demonstrated indicating that sequence similarities at selected housekeeping gene loci are poor markers of the overall similarity revealed by DNA-DNA hybridization. Nevertheless, a crude estimate by rarefaction analysis based on the examined sample of isolates suggests that the existing number of species according to current criteria (≥70% hybridization) that would belong in the red cluster is several hundreds. The limited hybridization data available for *S. oralis* and *S. infantis* cluster strains do not allow conclusions as to the number of distinct taxa at the species level within those clusters.

To estimate the evolutionary distance of *S. pneumoniae* strains and of strains in the remaining part of the pneumoniae-mitis-pseudopneumoniae cluster from a hypothetical immediate common ancestor we calculated the genetic distance of each strain from their common node identified by the Oralis cluster as an out-group in the tree (Arrow in Figure 1). The mean genetic distance of *S. pneumoniae* strains was 0.031±0.001 (Mean±Standard deviation) and the mean genetic distance of other strains of the pneumoniae-mitis-pseudopneumoniae cluster was 0.024±0.003. This difference is statistically highly significant (p<0.0001).

### Pneumococcus Virulence Gene Homologues in Commensal Relatives and Genome Reduction

**Figure 2:**
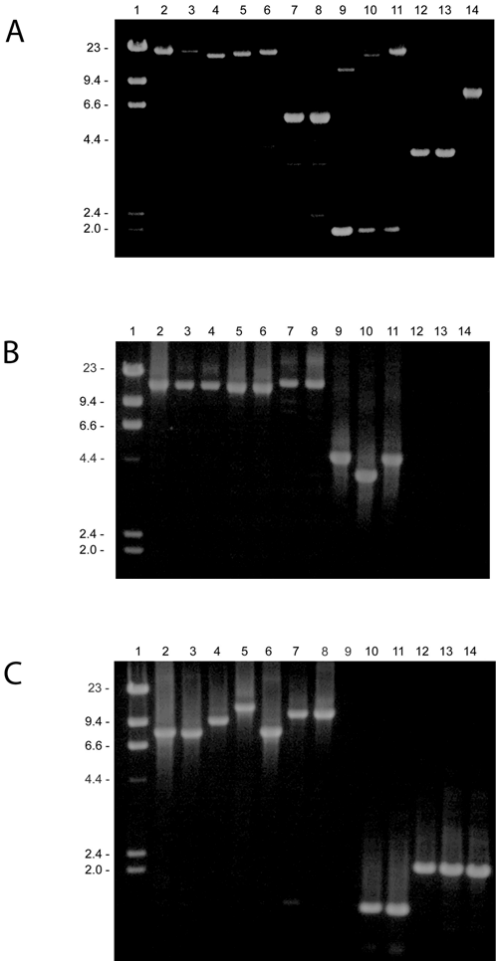
Examples of long range PCR for detection of virulence genes. Lane 1, molecular weight marker (sizes in kb are shown to the left); lanes 2–6, *S. pneumoniae* strains TIGR4, SK848, SK851, SK856, and SK858; lanes 7 and 8, *S. pseudopneumoniae* strains SK1069 and SK674; lanes 9–12, *S. mitis* strains SK575, SK568, SK142, and T2186; lanes 13 and 14, *S. oralis* strains SK23 and SK141. (A) PCR with primers *aliA* and *dexB* flanking the *cap* gene cluster. Amplicons larger than 7 kb represent the area between the genes *aliA* and *dexB*, whereas partial sequencing and control PCRs revealed that the smaller ones in lanes 7–13 are artifacts due to amplification of different regions with the *aliA* primer alone. The *S. pseudopneumoniae*, *S. mitis*, and *S. oralis* strains were selected to illustrate these artifacts. (B) and (C) Amplicons resulting from PCR with primers flanking the *ply*-*lytA* region and the *iga* gene, respectively.

Capsule production is a crucial virulence factor of *S. pneumoniae* and is generally assumed to distinguish pathogenic pneumococci from commensal Mitis group streptococci by its ability to confer protection against host phagocytes. In addition to capsule production, the significance of the IgA1 protease, pneumolysin, autolysin, neuraminidase, and several surface proteins has been demonstrated in experimental pneumococcal infections [7], [38]–[40]. During our previous analysis of cell-wall associated polysaccharides of *S. mitis* we unexpectedly identified a second polysaccharide distinct from the group O antigen [22]. As this polysaccharide may be the equivalent of the capsular polysaccharide in *S. pneumoniae* we analyzed the annotated nearly finished genome sequence of the type strain of *S. mitis* (JCVI-CMR). An operon flanked by the genes *dexB* and *aliA* and structurally similar to the *cap* operon in *S. pneumoniae* [41] was identified. This equivalent of a *cap* operon in *S. mitis* NCTC 12261 is composed of 20 genes and spans 21,357 bp (Figure S3). Among the 90 recognized capsular serotypes of *S. pneumoniae* a *cap* operon size range of 10,337 bp to 30,298 bp located between *dexB* and *aliA* was reported [41], whereas in certain pneumococci lacking capsule expression a *capN* homologue in addition to one or two open reading frames corresponding to the ABC transporter *aliB* and a variable number of Box elements amounting to 5 to 7 kb are present between *dexB* and *aliA* [42]. To determine the presence of a similar *cap* operon at this location we used long range PCR amplification in an examination of all 76 strains of the pneumoniae-mitis-pseudopneumoniae cluster. The primers reported by Bentley et al. [41] surprisingly did not amplify a product even in the *S. pneumoniae* strains except for strain TIGR4, which was included as a control. The alternative primer set subsequently designed for this study resulted in an amplicon from 8 strains (supplemented by 8 genome sequences) of *S. pneumoniae* and from 28 of the remaining 59 strains of the cluster. Fourteen of the non-pneumococcus amplicons were larger than 15 kb (the lower limit for *cap* operons in *S. pneumoniae* is 10 kb), and 13 were 5 to 7.5 kb (similar to non-encapsulated *S. pneumoniae*). Amplicons smaller than 6 kb obtained from some strains were shown by sequence analysis to be artifacts (Figure 2). It is conceivable that the 15–21 kb operons detected in half of the strains of the Mitis lineages (including the *S. mitis* type strain NCTC 12261 for which the sequence is available) encode proteins responsible for biosynthesis and transport of a capsular polysaccharide as exemplified by the one structurally identified in our previous study [22]. None of the three *S. pseudopneumoniae* had a *cap* operon of a size compatible with capsule expression. Among amplicons obtained from nine Oralis cluster strains three were suggestive of capsule expression. This is likely to represent the polysaccharide previously referred to as the coaggregation receptor carbohydrate in *S. oralis* by Cisar and his colleagues [43].

**Figure 3:**
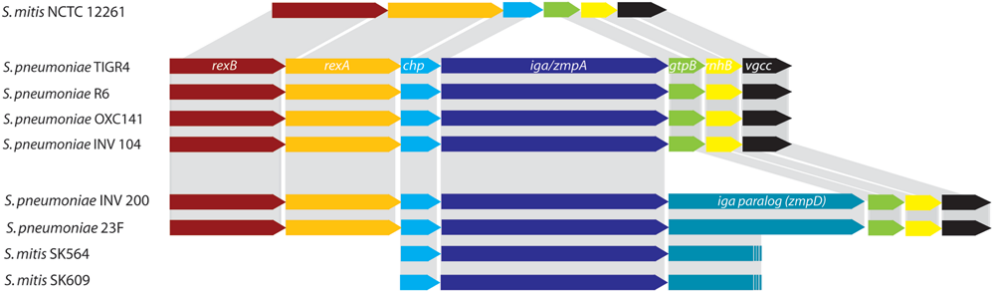
Map of *S. pneumoniae* genomes [8]–[44], [45] and the unfinished *S. mitis* genome (JCVI CMR) in the area that includes the IgA1 protease gene (*iga*). The map shows synteny apart from the absent *iga* gene in *S. mitis* NCTC 12261, in agreement with its lack of IgA1 protease activity, and apart from an additional *iga* paralog (*zmpD*) in some pneumococcus strains (INV200 and 23F) and in two strains of *S. mitis* [46].

Comparison of the *iga* gene locus in publicly available *S. pneumoniae* genomes (TIGR4, R6, OXC141, INV104, INV200, and 23F) with the corresponding area of the *S. mitis* type strain NCTC 12261 genome (www.tigr.org) showed complete synteny in the area containing the *iga* gene in *S. pneumoniae* apart from the absent *iga* gene in *S. mitis* NCTC 12261 (in agreement with phenotypic differences) and an additional *iga* paralog (*zmpD*) in some strains (Figure 3). PCR amplification of the area in all 57 strains of Mitis lineages and 17 *S. pneumoniae* strains resulted in amplicons from 25 strains. The size of the amplicon (larger than 7 kb or smaller than 2 kb) was in complete agreement with presence or absence of the demonstrated IgA1 protease activity and supports the conclusion that the *iga* gene, when present, is found in the same context as in *S. pneumoniae*. In contrast, all of five examined strains from the Oralis cluster gave an amplicon of approximately 2 kb in spite of detectable IgA1 protease activity, suggesting that the *iga* gene is located elsewhere in the *S. oralis* genome.

The pneumolysin gene (*ply*), once considered to be a defining property of *S. pneumonieae*, was recently demonstrated in occasional isolates of *S. mitis* [3], [47]. The primer pair used in the present study to detect *ply* and its neighboring gene *lytA*, which encodes autolysin, amplified a 15 kb fragment in *S. pneumoniae* strain TIGR4 and a 4.7 kb fragment from *S. mitis* strain SK142, of which the latter lacks these two virulence genes. The primers yielded a 15 kb amplicon in the majority of *S. pneumoniae* strains in accordance with presence of the *ply*-*lytA* region. Occasional absence of a PCR product was assumed to be due to lack of match of the primers. Most other strains of the pneumoniae-mitis-pseudopneumoniae cluster yielded a fragment of 4.7 or 4.0 kb indicating absence of the two genes. However, two of the *S. pseudopneumoniae* strains and three additional strains from the cluster yielded an amplicon compatible with the presence of both *ply* and *lytA* in the same region, and two strains gave an intermediate size amplicon (approximately 8 kb). Presence versus absence of the *ply-lytA* area was confirmed by Southern blotting using *ply* and *lytA* specific gene probes. This analysis demonstrated that the two strains yielding an intermediate size amplicon had the *lytA* gene but lacked *ply*. None of the strains in the Oralis and Infantis clusters showed evidence of *ply* or *lytA* except for the Mitis/Oralis hybrid strain (SK597), which possessed both.

Results of phenotypic characterization of the strains demonstrated that many metabolic enzymes as well as optochin susceptibility and bile solubility present in *S. pneumoniae* were present in lower proportions of other pneumoniae-mitis-pseudopneumoniae cluster strains (Table S2). Likewise, Southern blot analysis showed that 30% of strains of Mitis lineages but none of the strains of the Oralis and Infantis clusters showed presence of the *IS1381*, which has been considered characteristic of *S. pneumoniae*. BoxB elements, which are present in multiple copies interspersed in *S. pneumoniae* genomes and are capable of modulating gene expression, and enhance the genome plasticity [48], were present in all other pneumoniae-mitis-pseudopneumoniae cluster strains, though yielding band of varying numbers and intensities as detected by Southern blotting.

As shown in Figure 1 presence of *cap*, *iga*, *ply*, and *lytA* genes was randomly distributed in the strains belonging to the Mitis lineages of the red cluster in contrast to the *S. pneumoniae* in which we obtained evidence of their presence in the majority of strains. Combined with the identical location in the genome of these virulence genes, when present, the findings suggest a gradual loss of genes encoding virulence properties and selected metabolic enzymes, Box B elements, and *IS1381* in strains of the Mitis lineages.

### Genome size of *S. mitis* strains

To examine to what extent this gene loss affected the genome size of Mitis lineage strains we determined the approximate genome size of 10 strains. The size determined for the individual strains was SK135: 1,829 Kb, SK142/NCTC 12261: 1,832 Kb based on genome sequence, SK321: 1,686 Kb, SK322: 1,924 Kb, SK597: 1,777 Kb; SK599: 1,945 Kb, SK605: 1,668 Kb, SK609: 1,902 Kb, SK675: 1,626 Kb, and SK674: 1,866 Kb. In agreement with the genome reduction hypothesis the mean genome size of strains of the Mitis lineages was 1.8 mb (range: 1.7–1.9 mb) compared to a mean size of 2.1 mb for sequenced genomes of *S. pneumoniae* strains.

### Genome comparison of *S. pneumoniae* and *S. mitis*

Comparison of the genomes of *S. pneumoniae* strains TIGR4, R6, and G54 and the near finished genome of *S. mitis* NCTC 12261 using cut-off values of 40% amino acid sequence identity and ≥80% coverage of the query gene showed that 661, 457 and 451 proteins present in *S. pneumoniae* TIGR4, R6, and G54, respectively, were absent in the *S. mitis* type strain. When duplicate proteins shared by two or all three strains of *S. pneumoniae* and transposons, insertion sequences, and phage proteins were eliminated, a total of 765 proteins unique to *S. pneumoniae* relative to *S. mitis* remained. By comparison, 100 *S. mitis* proteins not present in any of the *S. pneumoniae* strains were identified when protein fragmentation due to the unfinished *S. mitis* genome was taken into account.

Blast search of each set of unique proteins revealed that 83 of the 100 “unique proteins” in *S. mitis* had homologs in *S. sanguinis*, *S. gordonii*, *S. agalactiae*, *S. thermophilus* and other related streptococci and none lacked homologs in other bacteria. In contrast, a total of 384 (excluding puplicates, transposons, insertion sequences, and phage proteins) of the 765 “unique proteins” in *S. pneumoniae* lacked known homologs outside *S. pneumoniae*. The vast majority of these 384 proteins are of unknown function and conceivably are proteins representing an ancestral form of the pneumococcus.

## Discussion

In spite of close genetic relationships, the pneumococcus and other members of the Mitis group of streptococci have strikingly different pathogenic potential. This difference is particularly striking for *S. pneumoniae* and *S. mitis*, which are genetically very closely related as evidenced by Figure 1. The goal of our phylogenetic analyses of this unique cluster of species was to elucidate the evolutionary processes that resulted in this situation.

The key question was if gene acquisition or gene loss explains the distinct pathogenic potential of the two species and how the immediate common ancestor of *S. pneumoniae* and *S. mitis* looked like. The sequence analyses as well as overall genome comparisons by DNA-DNA hybridization performed in the present study surprisingly demonstrated that *S. pneumoniae* is but one of several hundred distinct phylogenetic lineages of a cluster of otherwise commensal streptococci known as *S. mitis* or *S. pseudopneumoniae* in which *S. pneumoniae* is no more genetically divergent from other members of the cluster than individual lineages of *S. mitis* are from each other. The name *S. pseudopneumoniae* was recently assigned to one of these lineages [6]. This finding emphasizes that the evolutionary processes that determined the distinct pathogenic potential of *S. pneumoniae*, *S. pseudopneumoniae*, and the individual lineages of *S. mitis*, took place subsequent to the existence of a common ancestor of the three species after separation from other Mitis group streptococci such as *S. oralis* and *S. infantis*. This is also supported by the finding that selected orthologous genes in the pneumococcus-pseudopneumoniae-mitis cluster and the *S. oralis* cluster, respectively, were located in different contexts in the genome.

Theoretically, two different evolutionary scenarios may explain the difference in pathogenic potential. The phylogenetic tree in Figure 1 might imply that *S. pneumoniae* represents a lineage of the pneumoniae-mitis-pseudopneumoniae cluster that recently became a successful and rapidly expanding population. In this hypothetical scenario, its success and particular pathogenic potential could be a result of the successive import of virulence genes distributed among other lineages of the cluster as a result of individual acquisition from other sources. This scenario is in agreement with the general view that pathogenic bacteria evolve by acquisition of virulence properties [49]. However, several observations are in conflict with this explanation and support the opposite scenario, i.e., that, in recent times, commensal *S. mitis* lineages evolved from a pathogenic population as a result of loss of virulence and other genes. First, comparative analysis of three genomes of *S. pneumoniae* with that of the type strain of *S. mitis* showed significantly more proteins unique to three *S. pneumoniae* strains TIGR4, Rd, and G54 (N = 765) than to the *S. mitis* type strain (N = 100), although the latter figure conceivably is an underestimation due to the fact that only one genome sequence is available. The fact that 384 out of 765 unique *S. pneumoniae* proteins had no known homologs in other bacteria, strongly suggests an ancient origin of this lineage. In contrast, the finding that the majority of the significantly lower number of proteins unique to the *S. mitis* type strain relative to *S. pneumoniae* showed high homology to proteins in *S. sanguinis*, *S. gordonii* and other closely related streptococci and that none lacked homologs in other known bacteria suggest that the genes by which *S. mitis* differ from *S. pneumoniae* were acquired more recently, presumably independently by individual *S. mitis* lineages. Genome analysis of additional *S. mitis* strains is required to elucidate the latter hypothesis, but preliminary data obtained in our laboratory show distinct cell protein patterns of strains of *S. mitis* (not shown).

Secondly, support for the more ancient origin of the *S. pneumoniae* lineage was obtained by estimating genetic distances in the phylogenetic tree in Figure 1. These estimates showed that strains of *S. pneumoniae* are significantly more distant (p<0.0001) than strains of the *S. mitis* lineages from their common node (Arrow in Figure 1). The conclusion that this difference is in support of the more ancient origin of *S. pneumoniae* implies the same rate of diversification in the two populations, which is not necessarily true. Due to its different epidemiological behavior, *S. pneumoniae* is more likely to meet a diversity of potential recombination partners than commensal streptococci, which largely remain associated with single lineages of hosts. Although one result of homologous recombination is diversification, the process at high frequency may also contribute to homogenization of the gene pool, which conceivably is more prevalent for genes under no selection for diversity as evidenced by the low number of alleles of housekeeping genes relative to the total number of sequence types in *S. pneumoniae*. This may mean that the longer evolutionary distance calculated for *S. pneumoniae* may represent an underestimation.

Finally, the mosaic pattern of virulence-gene presence or absence in otherwise syntenic domains of the genomes of the commensal *S. mitis* lineages of the pneumoniae-mitis-pseudopneumoniae cluster, and the indirect evidence of gradual loss of selected phenotypic traits (Table S2) leading to significantly smaller genome size among the commensals support that gene loss has been and still may be an ongoing process in *S. mitis* lineages.

Based on this evidence we propose that the immediate common ancestor of the pneumoniae-mitis-pseudopneumoniae cluster was a bacterium with resemblance to the present-day pneumococcus with all the properties associated with virulence. One of these properties, the IgA1 protease, conceivably evolved by gene duplication in response to emergence of the immunoglobulin A1 (IgA1) subclass in the common ancestor of man, chimpanzees, and gorillas [50], which according to recent calculations existed 6 to 7 million years ago [51]. The IgA1 subclass became the principal mediator of adaptive immunity in the upper respiratory tract, the only habitat of these bacteria, and presumably exerted a strong selection pressure upon them. While the pneumococcus lineage conserved the expression of both capsule production and IgA1 protease activity to ensure their ability to colonize in the presence of IgA1 antibodies [52], lineages evolving into a commensal life style with a more subtle relationship with the mucosal immune system and the host in general gradually lost both characters and achieved the colonization advantage of the capsule-deficient phenotype [53]. The frequent isolation of capsule-deficient pneumococci may be an indication of the same process going on within that lineage, but at a slower rate. This evolutionary model proposing that the pneumoniae-mitis-pseudopneumoniae cluster arose from a pneumococcus-like organism pathogenic to the immediate ancestor of hominoids is consistent with our inability to isolate *S. mitis*-like bacteria from a range of mammals including old and new world monkeys, pigs, dogs, sheep, cattle, rats and mice (this study), while there is evidence of pneumococci causing infections in chimpanzees [54].

It is conceivable that the very small population of human individuals (100–1000) living some 120,000 years ago [55] represented a substantial bottleneck especially for pathogens like *S. pneumoniae* which, in contrast to commensals, induce immunity in their hosts resulting in discontinued colonization. The subsequent and very recent expansion of the human population facilitated the lineage-specific expansion of the pneumococcus population reflected in the phylogenetic tree in Figure 1.

In contrast to *S. pneumoniae*, the remaining part of the pneumoniae-mitis-pseudopneumoniae cluster diversified into numerous distinct lineages, which, in parallel with adaptation to a commensal life style with the ensuing loss of virulence genes and genome reduction, became sexually isolated as a result of the primarily vertical spread of commensal streptococci [56]. Simple physical isolation, stringent requirements for sequence similarity between donor and recipient for efficient homologous recombination, possibly combined with a lack of purging of genotypes from within the population as a result of immune tolerance towards commensal bacteria, resulted in genetic divergence to a degree that renders the majority of the high number of lineages separate species by current taxonomic standards. Although the consequence of identical or dissimilar competence stimulating peptides (CSP) for the ability of pairs of streptococci to exchange gene sequences is yet unknown, it is possible that the virtually strain-specific CSPs of these commensal streptococci contribute to the genetic barrier between individual lineages.

Several attempts have been made to identify crucial virulence factors of pneumococci in mouse models [38], [39]. Based on recognized relationships with humans, the results of the present study clearly demonstrate that capsule and IgA1 protease production in strains of some of the *S. mitis* lineages does not result in a pathogenic phenotype, suggesting that a multitude of properties working in concert make pneumococci pathogenic to man. The results of this study provide unique insight into the evolutionary history that resulted in genetically closely related streptococci with remarkably different pathogenic potential. Emergence of pathogenic bacteria is often explained by acquisition of key virulence genes by traditionally commensal species [49]. Our results suggest the opposite scenario, i.e., that commensal streptococci gradually evolved from a pathogen by genome reduction. The model finally provides an explanation to the difficulties often encountered in clinical microbiologic laboratories in differentiating *S. pneumoniae* and *S. mitis* and the findings challenge current definitions of bacterial species.

## Materials and Methods

### Bacterial strains and cultivation

The core of this study included 118 strains of α-hemolytic streptococci tentatively assigned to the species *S. pneumoniae*, *S. mitis*, *S. oralis, S. infantis*, and *S. pseudopneumoniae* based on comprehensive phenotypic characterization as described [21]. The strains were own isolates from healthy individuals, referred isolated from clinical microbiology laboratories and originating from infections including bacteremia in neutropenic patients and patients with endocarditis, and selected strains from reference culture collections. Some of the referred clinical isolates were received as potential intermediary forms of *S. pneumoniae* and *S. mitis* or *S. oralis*. Nine were isolated from Japanese individuals and the remaining isolates were from Caucasians (Table S3). An additional seven type strains of other species in the Mitis group of streptococci were included in selected analyses (See Figure S1). The bacteria were cultivated on Todd Hewitt agar (Difco Laboratories, Detroit, Mich.) incubated for 2 days at 37°C in an anaerobic chamber.

In an unsuccessful attempt to isolate *S. mitis* and *S. oralis*-like bacteria we sampled the buccal mucosa of a variety of mammals including new world monkeys, pigs, dogs, sheep, cattle, rats and mice. More than 100 isolates from Mitis salivarius agar cultures were phenotypically characterized as described [21] and sequencing of 16S rRNA was performed on selected strains.

### DNA sequencing

Internal fragments of the housekeeping genes *ddl*, *gdh*, *rpoB*, and *sodA* and of the 16S rRNA genes were amplified by PCR and sequenced using primers listed in Table S1. Primers purchased from DNA Technology (Aarhus, Denmark) were designed on the basis of published nucleotide sequences (Table S1). For the PCR we used approximately 1 ng whole-cell DNA as template and Ready-To-Go PCR beads (Amersham Pharmacia Biotech, Uppsala, Sweden). The temperature profile for the PCR was an initial denaturation at 94°C for 5 min, followed by 30 cycles at 94°C for 1 min, 60°C for 1 min, and 72°C for 2 min followed by a final extension at 72°C for 8 min. Amplicons were purified using Wizard Minicolumns (Promega, Madison, Wis.). Sequencing of both strands of the amplified fragments was achieved with the same primers and the Thermo Sequenase dye terminator cycle sequencing kit (Amersham Life Science, Cleveland, Ohio) with an Applied Biosystems PRISM 377 automated sequencer (Perkin-Elmer Applied Biosystems, Norwalk, Conn.).

We previously used sequences of *ddl*, *gdh*, *rpoB*, and *sodA* in a phylogenetic analysis of a broader spectrum of *Streptococcus* species [43]. While the ranges of the *rpoB* (516 nt) and *sodA* (366 nt) sequences was identical to those used previously the closer mutual relationship of the species examined in the present study made it possible to design alternative primers for *gdh* and *ddl* that resulted in longer sequences. The *ddl* (288 nt) and *gdh* (546 nt) sequences were partially overlapping with the sequences used in the MLST scheme for *S. pneumoniae*: ddl, nucleotides 577–864 (this study) versus 463–903 (MLST), and *gdh*, nucleotides 802–1347 (this study) versus 840–1299 (MLST).

Genomes of Mitis group streptococci include four copies of the rRNA gene operon, all present in the same orientation within the genome. For selected strains showing considerable ambiguity in areas of the 16S rRNA gene sequence, genome fragments each containing one copy of the16S rRNA gene were separated by pulsed field gel electrophoresis (PFGE) of genomic DNA prepared from bacteria harvested from a Todd-Hewitt broth culture [36] and cleaved in the agar plugs with 1 U of the intronic endonuclease I-*Ceu*I (New England Biolabs, Hertfordshire, United Kingdom), which recognizes a sequence unique to 23S rRNA genes [61]. This was possible because the four rRNA operons were in the same orientation in the genome. The individual fragments were purified from the gel using a Qiagen Gel Extraction kit (Quiagen) and subjected to amplification and sequencing of the 16S rRNA gene as described above. ComC sequences were determined as described previously [10] using primers listed in Table 1. Briefly, the primers 2tArg2 and 2tGlu, which are complementary to tRNA genes flanking the comCDE operon, were used to PCR-amplify a stretch of approximately 2.7 kb. The primer NPARG, which complements a sequence in the Arg-tRNA gene downstream of the 2tArg2 primer, was used for sequencing of the *comC* gene with the initial amplicon as template. The nucleotide sequences were translated to peptide sequences.

### Phylogenetic analysis

Phylogenetic and molecular evolutionary analyses were conducted using MEGA version 4.0 software [62]. The Minimum evolution algorithm was applied based on the Nucleotide Maximum Composite Likelihood analysis of all positions. Bootstrap analysis was based on 500 replicates. For comparison, analyses were also conducted with the Neighbor Joining algorithm.

### Detection of squences homologous to pneumococcus-associated virulence genes

The genome areas potentially containing the genes encoding proteins involved in capsular biosynthesis, IgA1 protease, pneumolysin, and autolysin were amplified in selected strains by long range PCR with primers designed on the basis of conserved sequences in flanking genes in genomes available for *S. pneumoniae* and in the unfinished *S. mitis* NCTC 12261 genome available at the J. Craig Venter Institute- Comprehensive Microbial Resource database (JCVI-CMR) (http://cmr.jcvi.org/tigr-scripts/CMR/CmrHomePage.cgi). For the long range PCR we used the Expand Long Template PCR System (Roche Molecular Biochemicals) with buffer 3, approximately 2 ng of whole-cell DNA as template, and 30 pmol of each primer in a 50 µl reaction volume. The thermal cycling program included denaturation for 2 min at 94°C, 30 cycles of 94°C for 10 sec, 55°C for 30 sec, and 68°C for 20 min, followed by an extension at 68°C for 10 min. The primers are listed in Table S4. For the *cps* gene cluster the two primers were based on conserved sequences in *dexB* (SP0342 in *S. pneumoniae* TIGR4) and *aliA* (SP0366 in *S. pneumoniae* TIGR4), which universally flank the *cap* operon in *S. pneumoniae* [41] and in *S. mitis* NCTC 12261 (JCVI-CMR). In addition, the oligonucleotides 1430 and 1402 described by Jiang et al. [63] were tried. For the *iga* gene area we used primers based on conserved sequences in a conserved hypothetical protein (SP1153 in *S. pneumoniae* TIGR4) and the ribonuclease H II gene (SP1156 in *S. pneumoniae* TIGR4), and for amplification of the *ply-lytA* region we used primers based on conserved sequences in the flanking *recA* (SP1940 in *S. pneumoniae* TIGR4) and the gene encoding an ABC transporter (SP1919 in *S. pneumoniae* TIGR4).

### Southern blot analysis

Presence of homologous gene sequences was confirmed by Southern blot analysis. The gene-specific probes represented the genes *ply*, *lytA*, IS*1381*, and BoxB in *S. pneumoniae* strain NCTC 7465^T^. Approximately 10 µg genomic DNA was digested with *Msp*I and *Eco*RI, respectively, according to the manufacturer's recommendations (Roche Molecular Biochemicals, Indianapolis, IN). Following treatment with 0.05 µg RNAse (DNase free, Roche Molecular Biochemicals) restriction fragments were separated by electrophoresis in 1% agarose gels for 16 h at 2 V/cm in TAE buffer (0.04 M Tris/acetate, 2 mM EDTA, pH 8.0). The separated fragments were transferred and fixed onto NytranN nylon membranes (Schleicher & Schuell, Keene, N.H.) and hybridizations were performed as described [64] except that the filters were soaked in 1% Triton X-100 before prehybridization and that 0.1% sodium pyrophosphate was included in all hybridization buffers. The DNA fragments used for probes were prepared by cloning PCR products into plasmid vector pCRII using the TA-Cloning kit (R&D Systems Europe Ltd., Abingdon, England). For the PCR amplifications performed as described above we used whole-cell DNA from *S. pneumoniae* strain NCTC7465^T^ as template and the primers listed in Table S4. The clonings were confirmed by partial DNA sequencing. The probe representing the gene encoding pneumolysin was a DNA fragment in the open reading frame encoding amino acids 8 to 449 as described by Walker et al. [65]. The probe for the *lytA* gene encoding the pneumococcal autolysin was a 624-bp fragment containing 181 nucleotides of the sequence upstream of the open reading frame and 443 nucleotides encoding the N-terminal part of the autolysin [66]. The probe representing insertion sequence IS*1381* was a 746-bp fragment of the 846-bp segment [67]. The oligonucleotide used as probe for the BOX element was 5′-GTCAGTTCTATCTACAACCTCAAAACAGTGTTTTGA-3′ representing the consensus sequence of the BoxB subunit [68]. The plasmid DNA probes were labeled with α^32^P-dATP (Amersham International, Amersham, England) by the Random Primed Labeling Kit (Roche Molecular Biochemicals). Hybridization was at 60°C and the final post-hybridization wash was at 60°C in 1×SSC (0.15 M NaCl, 0.015 M Na-citrate, pH 7.0), 0.1% SDS, 0.1% sodium pyrophosphate. Hybridization with an oligonucleotide labeled with γ^32^P-ATP (Amersham International) using T4 polynucleotide kinase (Roche Molecular Biochemicals) was performed in 6×SSC at 50°C as was the wash. The same membranes were used for all hybridizations each time after stripping by soaking in boiling 1% SDS. The *S. pneumoniae* TIGR4 genome harbours 127 BOX elements [8]. In Southern blots, *S. pneumoniae* strains showed 15 to 20 bands of varying intensity hybridizing with the BoxB probe. Therefore, a semi-quantitative estimate of BoxB elements in the genomes of strains (values 0–5+) was based on the number and intensity of hybridizing bands visible in the Southern blots.

### DNA-DNA Hybridization

DNA was isolated using a French pressure cell (Thermo Spectronic) and purified by chromatography on hydroxyapatite as described by Cashion *et al.* [69]. DNA-DNA hybridization was carried out at Deutsche Sammlung von Mikroorganismen und Zellkulturen Gmbh, Braunschweig, Germany as described by De Ley *et al.* [70], with the modifications described by Huss *et al.* [71] and Escara & Hutton [72], using a model 2600 spectrophotometer equipped with a model 2527-R thermo programmer and plotter (Gilford Instrument Laboratories).

### Phenotypic analyses

IgA1 protease, neuraminidase, and other biochemical activities were demonstrated as previously described [21], [36].

### Structure of cell wall polysaccharide

Two monoclonal antibodies, HAS and HASP8, specific for the phosphocholine residues and for the “backbone” of pneumococcal cell wall C-polysaccharide ( = Lancefield group O antigen), respectively, were used for examination of cell wall extracts by ELISA as described [22]. Each strain was propagated in 10 ml of Todd/Hewitt broth and harvested by centrifugation. The bacterial pellet was suspended in 1 ml lysis buffer consisting of 0.05 M Hepes, pH 7.0, 0.1 M NaCl, 1 mM MgCl_2_, 100 U mutanolysin and 1 mg lysozyme per ml (Sigma, St. Louis, MI, USA). After incubation for 4 h at 37°C cell debris was removed by centrifugation. The supernatant constituted the cell wall extract that was analyzed by ELISA as described [22].

### Coverage analysis

Coverage of existing species within the *S. mitis* complex of species was determined by rarefaction analysis using the program Analytic Rarefaction 1.3, available at http://www.uga.edu/strata/software/

### Genome size determination

Approximate genome sizes were determined for 9 strains of *S. mitis* (SK135, SK142/NCTC 12261, SK321, SK322, SK597; SK599, SK605, SK609, SK675) and one strain of *S. pseudopneumoniae* (SK674) by PFGE analysis of whole-cell DNA digested with *Sma*I. Digested genomic DNA from *S. pneumoniae* TIGR4 served as size reference. Genome sizes of *S. pneumoniae* strains for which the complete genome sequences are available and for *S. mitis* NCTC12261, for which a near complete genome sequence is available, were extracted from the respective public databases.

### Comparative estimation of the number of unique genes

Genomes of *S. pneumoniae* strains TIGR4, R6, and G54 and *S. mitis* NCTC 12261 were compared to identify genes that were unique to *S. pneumoniae* and *S. mitis*, respectively. The search was an NCBI blastp of each polypeptide against the non-redundant database. The search used an expectation value cutoff of 1e-10 and BLAST was instructed to report the top 1000 hits. The resulting HSPs were filtered to include only those that contributed to the best sum(p) for that particular query/target pair. These HSPs were aggregated to eliminate overlap, where necessary, and the average % Identity and % Coverage of the query molecule was calculated. These hits were then filtered at 40% identity and 80% coverage. The source organism for all hits was retrieved from the NCBI and hits were grouped by query protein and source organism to produce a list of organisms and the number of mitis/pneumo unique genes that hit each.

## Supporting Information

Figure S1 Phylogenetic tree constructed with the minimal evolution algorithm and based on partial 16S rRNA gene sequences of 80 strains of mitis group streptococci. Type strains are shown with species designation. The settings in the program MEGA 3.1 were as follows: Gaps/missing: pairwise deletions; Distance method: Nucleotide: Tamura and Nei (Gamma = 1). Bootstrap values (%) are based on 1000 replications (only values above 70% are shown). Note dual alleles harbored by four strains are included separately (alleles designated A and B). Strains allocated to species/ clusters according to phylogenetic analysis based on concatenated sequences of housekeeping genes (Figure 1) are indicated by green, Infantis cluster; ruby, *S. pneumoniae*, pink, *S. pseudopneumoniae*, red, *S. mitis*; blue, Oralis cluster.
(11.79 MB TIF)

Figure S2 Phylogenetic tree constructed with the minimum evolution algorithm and based on partial sequences of individual 16S rRNA genes (439 to 449 nucleotides corresponding to positions 51 to 493 in the *Escherichia coli* 16S rRNA gene in 4 strains showing sequence polymorphism among the four individual rRNA operons labeled A through D. Sequences obtained for type strains of *S. mitis* and *S. oralis* are included for comparison. The numbering of nucleotides is according to the 16S rRNA gene of *E. coli*. Bootstrap values (%) are based on 1000 replicates. Gaps are indicated by “-”
(2.36 MB TIF)

Figure S3 Gene organization of the cap locus between *dexB* and *aliA* and flanking regions in *S. mitis* NCTC12261 compared with *S. pneumoniae* strains D39, G54, and TIGR4.
(26.13 MB TIF)

Table S1 Amino acid sequence of competence stimulating peptides (CSPs) from strains of streptococci assigned to species according to the cluster analysis in Fig. 1 (SK strains) or as indicated in previously publications. Cluster-specific amino acid signatures within the leader sequence are summarized in Table 1.
(0.11 MB DOC)

Table S2 Phenotypic properties and BoxB and insertion sequence elements among *S. pneumoniae*, *S. pseudopneumoniae*, and *S. mitis* strains illustrating genome reduction in *S. mitis*
(0.04 MB DOC)

Table S3 Strains analyzed in the study with site of isolation, origin, and previous designation.
(0.29 MB DOC)

Table S4 Primers used for PCR amplification and sequencing of genes.
(0.03 MB DOC)
