# Correction: Evolution of *Streptococcus pneumoniae* and Its Close Commensal Relatives

**DOI:** 10.1371/annotation/0e3332aa-1b10-4e1a-a424-546d2cb7cfff

**Published:** 2009-12-11

**Authors:** Mogens Kilian, Knud Poulsen, Trinelise Blomqvist, Leiv S. Håvarstein, Malene Bek-Thomsen, Hervé Tettelin, Uffe B. S. Sørensen

Figure S3 contains errors. Please view the corrected file here: 
