# Supplementary figures and images for: Evolution of Streptococcus pneumoniae and Its Close Commensal Relatives
Source: PLoS One. 2008 Jul 16;3(7):e2683. doi: 10.1371/journal.pone.0002683 (PMC2444020; doi:10.1371/journal.pone.0002683)

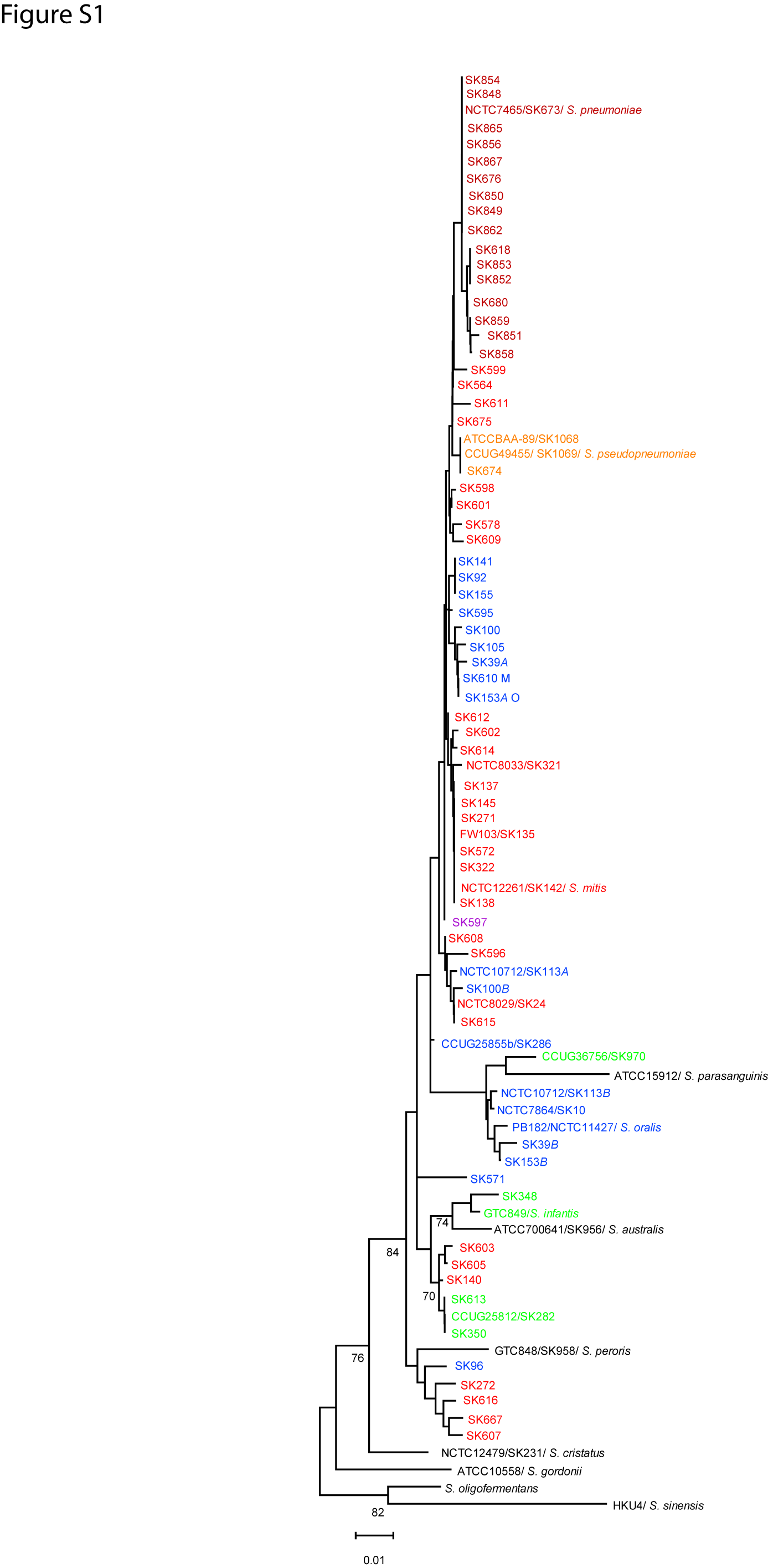

Supplement: Figure S1 — Phylogenetic tree constructed with the minimal evolution algorithm and based on partial 16S rRNA gene sequences of 80 strains of mitis group streptococci. Type strains are shown with species designation. The settings in the program MEGA 3.1 were as follows: Gaps/missing: pairwise deletions; Distance method: Nucleotide: Tamura and Nei (Gamma = 1). Bootstrap values (%) are based on 1000 replications (only values above 70% are shown). Note dual alleles harbored by four strains are included separately (alleles designated A and B). Strains allocated to species/ clusters according to phylogenetic analysis based on concatenated sequences of housekeeping genes (Figure 1) are indicated by green, Infantis cluster; ruby, S. pneumoniae, pink, S. pseudopneumoniae, red, S. mitis; blue, Oralis cluster. (11.79 MB TIF) [file pone.0002683.s001.tif]

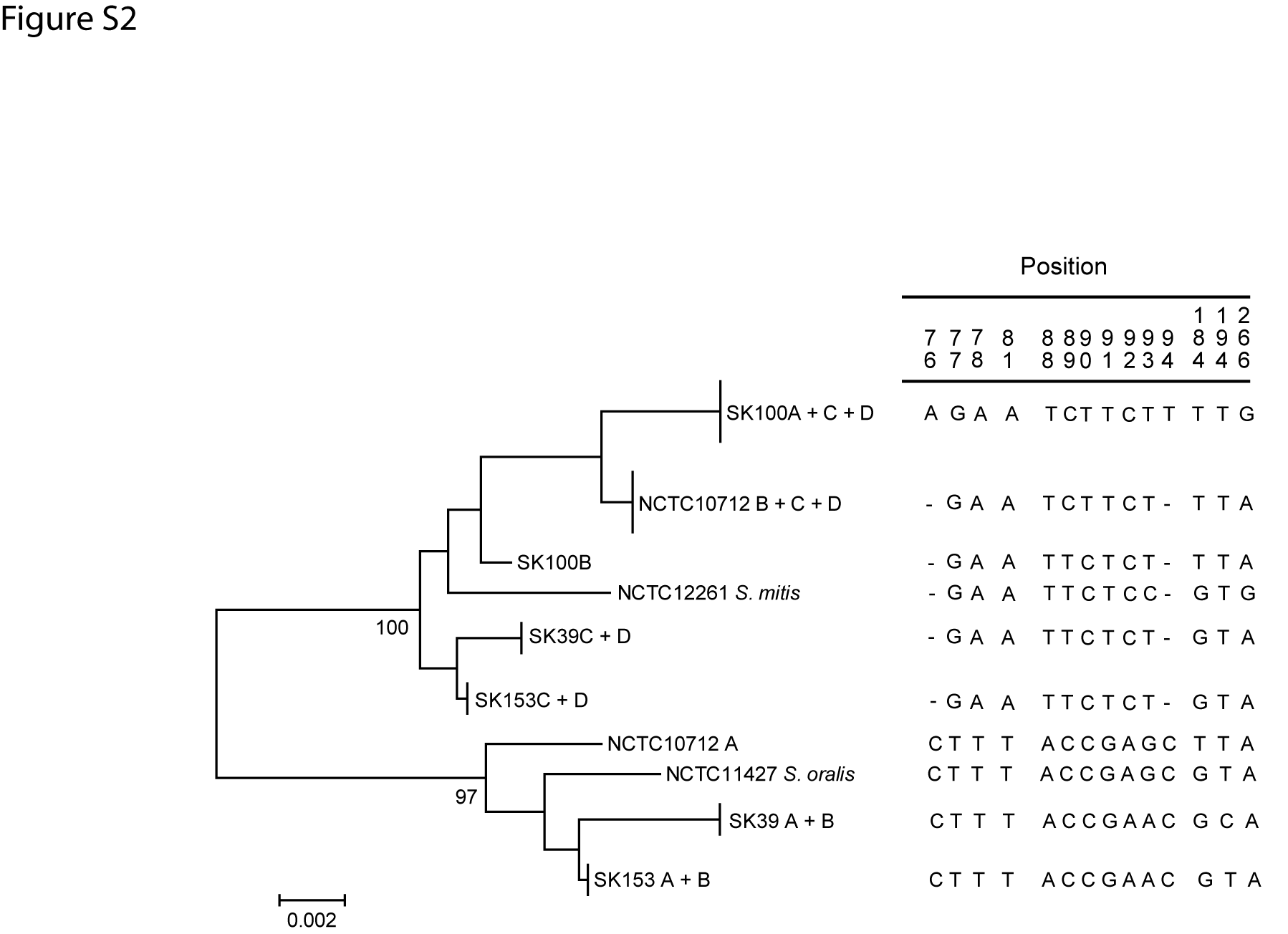

Supplement: Figure S2 — Phylogenetic tree constructed with the minimum evolution algorithm and based on partial sequences of individual 16S rRNA genes (439 to 449 nucleotides corresponding to positions 51 to 493 in the Escherichia coli 16S rRNA gene in 4 strains showing sequence polymorphism among the four individual rRNA operons labeled A through D. Sequences obtained for type strains of S. mitis and S. oralis are included for comparison. The numbering of nucleotides is according to the 16S rRNA gene of E. coli. Bootstrap values (%) are based on 1000 replicates. Gaps are indicated by “-” (2.36 MB TIF) [file pone.0002683.s002.tif]

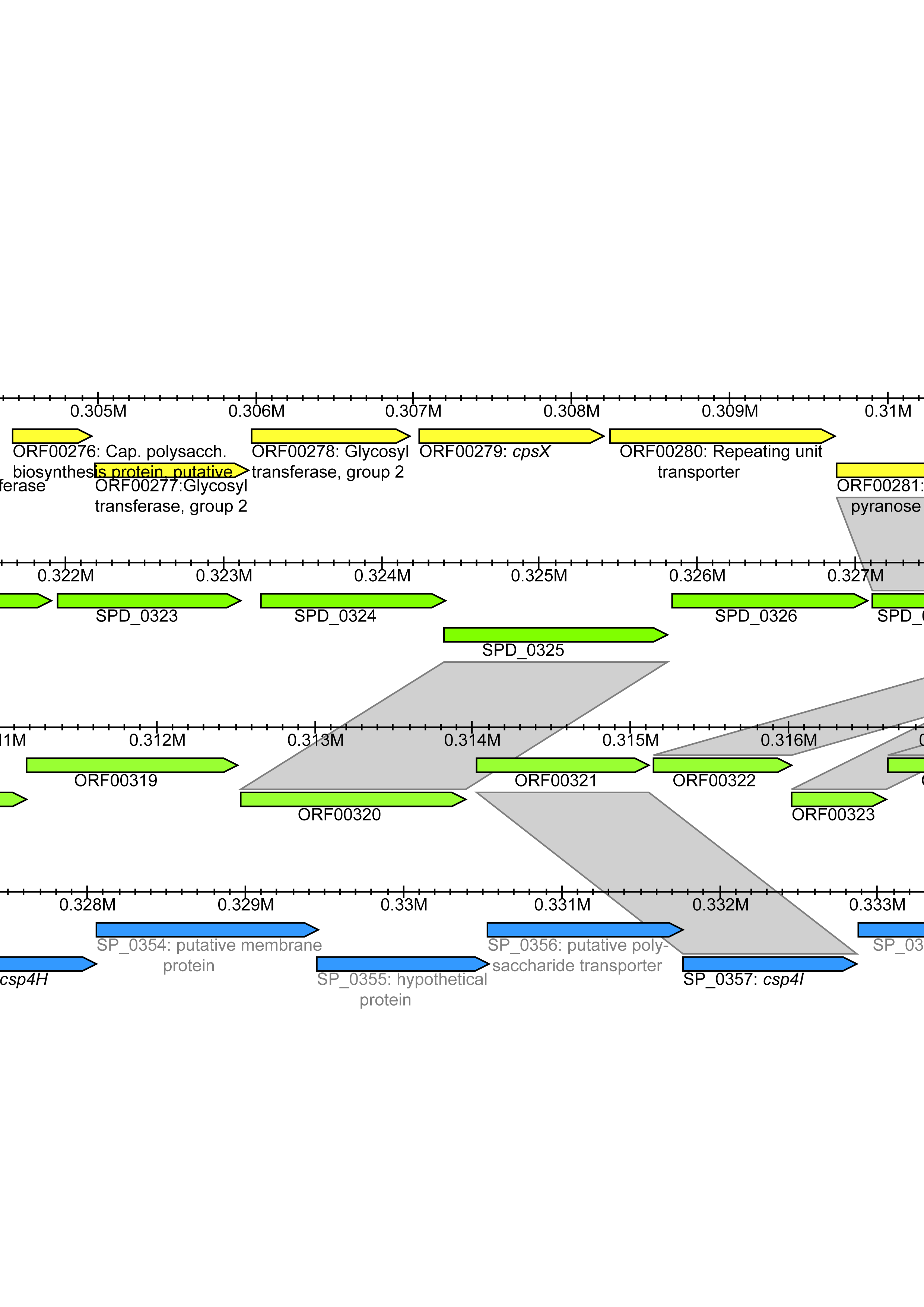

Supplement: Figure S3 — Gene organization of the cap locus between dexB and aliA and flanking regions in S. mitis NCTC12261 compared with S. pneumoniae strains D39, G54, and TIGR4. (26.13 MB TIF) [file pone.0002683.s003.tif]
