# Supplementary material for: Evolution of Streptococcus pneumoniae and Its Close Commensal Relatives
Source: PLoS One. 2008 Jul 16;3(7):e2683. doi: 10.1371/journal.pone.0002683 (PMC2444020; doi:10.1371/journal.pone.0002683)
Supplement: Table S1 — Amino acid sequence of competence stimulating peptides (CSPs) from strains of streptococci assigned to species according to the cluster analysis in Fig. 1 (SK strains) or as indicated in previously publications. Cluster-specific amino acid signatures within the leader sequence are summarized in Table 1. (0.11 MB DOC) [file pone.0002683.s004.doc]

**Table S1.** Amino acid sequence of competence stimulating peptides (CSPs) from strains of streptococci assigned to species according to the cluster analysis in Fig. 1 (SK strains) or as indicated in previously publications. Cluster-specific amino acid signatures within the leader sequence are summarized in Table 1.

| **Species**  **Strain** | Ref. | Amino acid sequence of ComC **Sequence of mature CSP is given in bold capital letters** |
| --- | --- | --- |
| ***S. pneumoniae*** |  |  |
| Rx | [29] | mkntvkleqfvalkekdlqkikgg**EMRLSKFFRDFILQRKK** |
| NCTC 7465^T^/SK673 |  |
| SK 848 |  |
| SK 852 |  |
| SK 853 |  |
| SK 856 |  |
| SK 858 |  |
| SK 862 |  |
| SK 865 |  |
| SK 867 |  |
| A66 | [30] | mkntvkleqfvalkekdlqkikgg**EMRISRIILDFLFLRKK** |
| SK 618 |  |
| SK 680 |  |
| SK 851 |  |
| SK 854 |  |
| CSP-2.2 | [31] | mkntvkleqfvalkekdlqnikgg**EMRISRIILDFLFLRKK** |
| SK 676 |  | mkntvkleqfkkltekelqeiqgg**ERRIPDVIRSLLFQKRK** |
| CSP-3 | [31] | mkntvkleqfvalkekdlqniqgg**EMRKMNEKSFNIFNFFNFFNFFRRR** |
| CSP-4 | [31] | mkntvkleqfvalkekdlqniqgg**EMRKMNEKSFNIFNFFNFFRRR** |
| CSP-5 | [31] | mkntvkleqfvdlkekdlqeiqgg**ESRLPKILLDFLFLRKK** |
| CSP-6.1 | [31] | mkntvkleqfvalkekdlqkikgg**EMRLPKILRDFIFPRKK** |
| *S. mitis* |  |  |
| B5 | [32] | mkntvkleqfvalkekdlqkikgg**ESRLPKIRFDFIFPRKK** |
| B6 | [32] | mkntvkleqfvalkekdlqkikgg**EMRKPDGALFNLFRRR** |
| Hu8 | [32] | mkntvkleqfvalkekdlqkiqgg**EMRKSNNNFFHFLRRI** |
| SK 262 |  | mkntvkleqfvalkekdlqkiqgg**EMRRSNNNFFNFLRRI** |
| CSP-7 | [31] | mkntvkleqfvalkekdlqeirgg**ESRVSRIILDFLFQRKK** |
| CSP-8 | [31] | mkntvkleqfvalkekdlqnikgg**ESRISDILLDFLFQRKK** |
| SK 675 |  | mkntvkleqfvalkekdlqeirgg**ESRVSRIILDFLFLRKK** |
| SK 612 |  | mkntvkleqfvalkekdlqkikgg**ESRLSRLLRDFIFQIKQ** |
| SK 611 |  | mkntvkleqfvalkekdlqeikgg**EMRLPKILRDFIFPRKK** |
| SK 564 |  | mknivkleqfvvlkekdlqkiqgg**ESRVSDILLDFLFRRKK** |
| SK 137 |  | mkntvkleqfvalkekdlqeiqgg**ESRLPKIRFDFIFPRKK** |
| SK 615 |  | mkntvkleqfvalkekdlqkiqgg**EMRKSNNTFFNFLRRI** |
| SK 598 |  | mkntvkleqfvalkekdlknikgg**EMRRIDKIFINFLKRR** |
| SK272 |  | mkntvkleqfvalkekdlgniqgg**EMRRIDKIFINFLKRR** |
| SK616 |  |
| SK 599 |  | mkntvkleqfvalkekdlqeikgg**EMRRIGSVLLNFFKRR** |
| NCTC 8029/CSP-9 | [32] | mkntvkleqfvalkekdlqkiqgg**EMRKPDGALFNLFRRR** |
| SK 596 |  | mkntvkleqfvslkekdlqkiqgg**EMRKPDGALFILFRRR** |
| SK 601 |  | mkntvqleqfvalkekdlqkikgg**EMRKMNEKSFNIFNIFSIFRRR** |
| SK 614 |  | mkntvkleqfvalkekdlqkikgg**EMRKMNEKSFNFFNIFRRR** |
| SK 667 |  | mkntvkleqfvslkekdlqkiqgg**EIRKSNSALVNFFKRR** |
| SK 609 |  | mkntvkleqfvdlkekdlqkikgg**EIRKTSNSLLNFFKRR** |
| SK 608 |  | mkntvkleqfvalkekdlqkingg**EIRKTSNSLLNFFKRR** |
| SK 602 |  | mkntakleqfvalkekdlqkikgg**EIRKTSNSLLNFFKRR** |
| NTCT 12261^T^ | [32] | mkntvnldkfvelkekdlqniqgg**EIRQTHNIFFNFFKRR** |
| SK 138 |  |
| SK 271 |  |
| SK 572 |  |
| SK 322 |  |
| SK 145 |  | mkntvkleqfveltevemqeiqgg**DMRISESIRNLIFPRKKK** |
| *S.pseudopneumoniae* |  |  |
| CCUG49455/SK1069 |  | mkntvkleqfvalkekdlqkikgg**EMRLPKILRDFIFPRKK** |
| SK 674 |  | mkkntdfaqmkdfqqlnekelqeirgg**EWRPPYTINNFLFPKRK** |
| *S. oralis* |  |  |
| SK23/NCTC 11427 ^T^ | [32] | mkntekleqfkevteaelqeirgg**DKRLPYFFKHLFSNRTK** |
| SK10/NCTC 7864 |  |
| SK 595 |  |
| SK 610 |  | mkntgkleqfkevteaelqeirgg**DKRLPYFFKHLFSNRTK** |
| SK153 |  | mkntvkleqfkevteaelqeirgg**DKRLPYFFKHLFSNRTK** |
| SK 286 |  |
| SK 39 |  | mkntvkleqfkkltekelqeiqgg**DKRGLMDLFKQIPIFRRK** |
| SK79 |  |
| SK 571 |  | mnntvqleqfkevtetelqeirgg**DKRGLMDLFKQIPIFRRK** |
| SK 92 |  | mkntvkleqfkevteaelqeirgg**DWRISETIRNLIFPRRK** |
| SK 100 |  |
| SK 105 |  |
| SK 113 |  |
| SK 141 |  |
| SK 155 |  |
| SK 304 |  |
| DSM 20066 |  |  |
| SK 34 |  | mkntvkleqfkevtetelqeirgg**EWRIPELIRNLIFPKRK** |
| SK96 |  |
| SK103 |  |
| SK 305 |  | mkntekleqfkkvteaelqeirgg**EIRKENNFLFYFFKRK** |
| CSP-6.2 | [31] | mkntvkleqfvalkekdlqeikgg**EMRLPKILRDFIFPRKK** |
| SK 597^1^ |  | mkntvkleqfvalkekdlqkirgg**EMRRIDEKLVGIFNFFRRR** |
| ***S. infantis*** |  |  |
| SK 140 |  | mkkntqfaqmkdfqelnekelqeirgg**DIRLPHIIKKLFSK** |
| SK 350 |  | mkkhtgfaqmkdfqelnekelqeirgg**EWRPMYTINNFLFSKSK** |
| SK 283 |  | mkkntgfaqmkdfqqlnekelqeirgg**DIRSFKFLNKIFPKK** |
| SK 282/ CCUG25812 |  | mkkntdfaqmkdfqqlnekelqeirgg**DIRSFKFLNKIFPKK** |
| SK 605 |  |

^1^According to other data (see text) SK597 is a hybrid of *S. mitis* and *S. oralis*.
