# Supplementary material for: Evolution of Streptococcus pneumoniae and Its Close Commensal Relatives
Source: PLoS One. 2008 Jul 16;3(7):e2683. doi: 10.1371/journal.pone.0002683 (PMC2444020; doi:10.1371/journal.pone.0002683)
Supplement: Table S2 — Phenotypic properties and BoxB and insertion sequence elements among S. pneumoniae, S. pseudopneumoniae, and S. mitis strains illustrating genome reduction in S. mitis (0.04 MB DOC) [file pone.0002683.s005.doc]

**Table S2**. Phenotypic properties and BoxB and insertion sequence elements among *S. pneumoniae, S. pseudopneumoniae*, and *S. mitis* strains illustrating genome reduction in *S. mitis*

|  | Percent positive reactions in individual taxa | | |
| --- | --- | --- | --- |
|  | *S. pneumoniae* | *S. pseudopneumoniae* | *S. mitis* |
| Character^1^ | N = 17 | N = 3 | N = 54 |
| Arginine dihydrolase (R) | 29 | 0 | 0 |
| Aesculine hydrolysis (T) | 47 | 0 | 7 |
| Hyaluronidase | 82 | 0 | 0 |
| IgA1 protease | 100 | 100 | 55 |
| Optochine susceptibility | 100 | 67 | 4 |
| Bile solubility | 94 | 33 | 7 |
| Neuraminidase | 100 | 100 | 69 |
| Fermentation of: |  |  |  |
| Cellobiose (T) | 65 | 0 | 22 |
| Glycogen (T) | 82 | 0 | 43 |
| Inuline (T) | 71 | 33 | 4 |
| Melibiose (T) | 82 | 33 | 27 |
| Raffinose (R) | 53 | 33 | 22 |
| Trehalose (R) | 71 | 0 | 4 |
| Production of: |  |  |  |
| α-galactosidase (R) | 88 | 33 | 27 |
| N-acetyl-β-glucosaminidase (R) | 53 | 0 | 7 |
| Pyrolidonyl arylamidase (T) | 94 | 0 | 0 |
| Pyroglutamic acid arylamidase (R) | 65 | 0 | 4 |
| Genetic elements: |  |  |  |
| BoxB elements | 4+-5+ | 4+-5+ | 3+-5+ |
| IS*1381* | 100 | 33 | 30 |

^1^Biochemical tests performed by traditional tube tests (T) or in the rapid ID 32 STREP kit (R) as described in the text.
