# Supplementary material for: Evolution of Streptococcus pneumoniae and Its Close Commensal Relatives
Source: PLoS One. 2008 Jul 16;3(7):e2683. doi: 10.1371/journal.pone.0002683 (PMC2444020; doi:10.1371/journal.pone.0002683)
Supplement: Table S3 — Strains analyzed in the study with site of isolation, origin, and previous designation. (0.29 MB DOC) [file pone.0002683.s006.doc]

**Table S3.**  Strains analyzed in the study with site of isolation, origin, and previous designation.

| Strain number | Alternative designation | Species according to this study | Previous designation | Isolated from | Origin | Recieved from^1^ | Reference |
| --- | --- | --- | --- | --- | --- | --- | --- |
| SK10 | NCTC 7864 | *S. oralis* | *S. sanguinis* |  |  | NCTC |  |
| SK23 | NCTC 11427 | *S. oralis* T |  | Human mouth | Sweden | Sneath | [57] |
| SK24 | NCTC 8029 | *S. mitis* | *S. oralis* |  | U.K. | NCTC |  |
| SK34 | CCUG 25611 | *S. oralis* | *S. mitis* biovar 2 | Human mouth | Denmark | Own isolate | [21] |
| SK39 | CCUG 55088 | *S. oralis* |  | Human mouth | Denmark | Own isolate | [21] |
| SK79 |  | *S. oralis* | *S. mitis* biovar 2 | Human mouth | Denmark | Own isolate | [21] |
| SK92 |  | *S. oralis* |  | Dental plaque | Denmark | Own isolate | [21] |
| SK95 |  | *S. oralis* | *S. mitis* biovar 2 | Human mouth | Denmark | Own isolate | [21] |
| SK96 |  | *S. oralis* |  | Human mouth | Denmark | Own isolate | [21] |
| SK100 |  | *S. oralis* |  | Dental plaque | Denmark | Own isolate | [21] |
| SK103 |  | *S. oralis* | *S. mitis* biovar 2 | Human mouth | Denmark | Own isolate | [21] |
| SK105 |  | *S. oralis* |  | Human mouth | Denmark | Own isolate | [21] |
| SK113 | NCTC 10712 | *S. oralis* | *Streptococcus* sp., viridans type (?) |  | U.K. | NCTC |  |
| SK135 | CCUG 55079/ FW103 | *S. mitis* | *Streptococcus* sp. V:A | Human mouth | Sweden | Carlsson | [58] |
| SK137 | CCUG 55080 | *S. mitis* |  | Dental plaque | Denmark | Own isolate | [21] |
| SK138 | CCUG 55081 | *S. mitis* |  | Dental plaque | Denmark | Own isolate | [21] |
| SK140 | HPA1 | *S. infantis* | *Streptococcus* sp. I:A | Human mouth | Sweden | Carlsson | [59] |
| SK141 |  | *S. oralis* |  | Dental plaque | Denmark | Own isolate | [21] |
| SK142 | NCTC12261/NS51 | *S. mitis* T |  | Human mouth | Sweden | NCTC | [58] |
| SK145 | OS-51 | *S. mitis* |  | Human mouth | Sweden | Carlsson | [58] |
| SK153 | 178/80 | *S. oralis* |  | Human blood | Denmark | SSI | [21] |
| SK155 | CCUG 55091/ 950/80 | *S. oralis* |  | Human blood | Denmark | SSI | [21] |
| SK262 |  | *S. mitis* |  | Human mouth | Sweden | Own isolate |  |
| SK271 |  | *S. mitis* |  | Human mouth | Denmark | Own isolate |  |
| SK272 |  | *S. mitis* |  | Human mouth | Sweden | Own isolate |  |
| SK282 | CCUG 25812 | *S. infantis* | *S. mitis* | CAPD fluid | Sweden | CCUG |  |
| SK283 | CCUG 25857 | *S. infantis* | *S. pseudomilleri* | Clinical specimen | Sweden | CCUG |  |
| SK286 | CCUG 25855b | *S. oralis* |  |  |  | CCUG |  |
| SK304 | H1 | *S. oralis* |  |  | U.S.A. | Cisar |  |
| SK305 | J22 | *S. oralis* |  |  | U.S.A. | Cisar |  |
| SK319 | ATCC 12398 | *S. mitis* | *Streptococcus* sp. Group O |  | U.K. | ATCC |  |
| SK320 | NCTC 8031 | *S. mitis* | *Lactococcus lactis subsp. lactis* |  | U.K. | NCTC |  |
| SK321 | NCTC 8033 | *S. mitis* | *Lactococcus lactis subsp. lactis* |  | U.K. | NCTC |  |
| SK322 |  | *S. mitis* |  | Human mouth | Denmark | Own isolate |  |
| SK350 | 22A | *S. infantis* |  | Human mouth | U.S.A. | Brown |  |
| SK556 |  | *S. mitis* |  | Human blood | Denmark | KMA, Aarhus |  |
| SK562 |  | *S. oralis* |  | Endocarditis | Denmark | KMA, Aarhus |  |
| SK564 |  | *S. mitis* |  | Human blood | Denmark | KMA, Aarhus |  |
| SK565 |  | *S. oralis* |  | Human blood | Denmark | KMA, Aarhus |  |
| SK568 | H452 | *S. mitis* |  | Blood, neutropenic | Switzerland | Francioli |  |
| SK569 | H2112 | *S. mitis* |  | Blood, neutropenic | Switzerland | Francioli |  |
| SK571 | H4268 | *S. oralis* |  | Blood, neutropenic | Switzerland | Francioli |  |
| SK572 | H4000 | *S. mitis* |  | Blood, neutropenic | Switzerland | Francioli |  |
| SK573 | H5073 | *S. oralis* |  | Blood, neutropenic | Switzerland | Francioli |  |
| SK575 | H5422b | *S. mitis* |  | Blood, neutropenic | Switzerland | Francioli |  |
| SK576 | H7638a | *S. oralis* |  | Blood, neutropenic | Switzerland | Francioli |  |
| SK577 | H7638c | *S. oralis* |  | Blood, neutropenic | Switzerland | Francioli |  |
| SK578 | H9883 | *S. mitis* |  | Blood, neutropenic | Switzerland | Francioli |  |
| SK580 | 60469-1 | *S. oralis* |  | Human blood | Denmark | KMA, Aarhus |  |
| SK581 | 60469-3 | *S. oralis* |  | Human blood | Denmark | KMA, Aarhus |  |
| SK595 | CCUG 55093 | *S. oralis* |  | Human blood | Denmark | SSI |  |
| SK596 |  | *S. mitis* |  |  | Denmark | SSI |  |
| SK597 | CCUG 55094 | *S. mitis/oralis* hybrid |  | Human urethra | Denmark | SSI |  |
| SK598 |  | *S. mitis* |  |  | Denmark | SSI |  |
| SK599 |  | *S. mitis* |  | Pharynx, atopic child |  | Own isolate | [59] |
| SK601 | CCUG 55084 | *S. mitis* |  | Pharynx, atopic child |  | Own isolate | [59] |
| SK602 |  | *S. mitis* |  | Pharynx, child |  | Own isolate | [59] |
| SK603 |  | *S. infantis* |  | Pharynx, child |  | Own isolate | [59] |
| SK605 |  | *S. infantis* |  | Pharynx, atopic child |  | Own isolate | [59] |
| SK607 |  | *S. mitis* |  | Pharynx, atopic child |  | Own isolate | [59] |
| SK608 | CCUG 55085 | *S. mitis* |  | Pharynx, child |  | Own isolate | [59] |
| SK609 |  | *S. mitis* |  | Pharynx, atopic child |  | Own isolate | [59] |
| SK610 |  | *S. oralis* |  | Pharynx, atopic child |  | Own isolate | [59] |
| SK611 |  | *S. mitis* |  | Pharynx, atopic child |  | Own isolate | [59] |
| SK612 | CCUG 55086 | *S. mitis* |  | Pharynx, atopic child |  | Own isolate | [59] |
| SK613 | 7207/95 | *S. infantis* |  |  | Denmark | SSI |  |
| SK614 | 535/91 | *S. mitis* |  |  | Denmark | SSI |  |
| SK615 | 997/95 | *S. mitis* |  |  | Denmark | SSI |  |
| SK616 | 6680/95 | *S. mitis* |  |  | Denmark | SSI |  |
| SK618 | Vac.st.45 | *S. pneumoniae* serotype 45 |  |  | Denmark | SSI |  |
| SK623 |  | *S. mitis* |  | Mouth, child | Denmark | Own isolate | [56] |
| SK626 |  | *S. mitis* |  | Mouth, child | Denmark | Own isolate | [56] |
| SK627 |  | *S. mitis* |  | Mouth, child | Denmark | Own isolate | [56] |
| SK632 |  | *S. mitis* |  | Mouth, child | Denmark | Own isolate | [56] |
| SK634 |  | *S. mitis* |  | Mouth, child | Denmark | Own isolate | [56] |
| SK635 |  | *S. mitis* |  | Mouth, child | Denmark | Own isolate | [56] |
| SK637 |  | *S. mitis* |  | Mouth, child | Denmark | Own isolate | [56] |
| SK640 |  | *S. mitis* |  | Mouth, child | Denmark | Own isolate | [56] |
| SK646 |  | *S. infantis* |  | Mouth, child | Denmark | Own isolate | [56] |
| SK648 |  | *S. mitis* |  | Mouth, child | Denmark | Own isolate | [56] |
| SK651 |  | *S. mitis* |  | Mouth, child | Denmark | Own isolate | [56] |
| SK653 |  | *S. mitis* |  | Mouth, child | Denmark | Own isolate | [56] |
| SK656 |  | *S. infantis* |  | Mouth, child | Denmark | Own isolate | [56] |
| SK659 |  | *S. mitis* |  | Mouth, child | Denmark | Own isolate | [56] |
| SK661 |  | *S. mitis* |  | Mouth, child | Denmark | Own isolate | [56] |
| SK667 |  | *S. mitis* |  | Meningitis | Denmark | KMA, Aarhus |  |
| SK673 | NCTC 7465 | *S. pneumoniae* T serotype 1 |  |  | U.S.A. | NCTC |  |
| SK674 |  | *S. pseudopneumoniae* |  | Human throat | Denmark | Own isolate |  |
| SK675 |  | *S. mitis* |  | Human throat | Denmark | Own isolate |  |
| SK676 |  | *S. pneumoniae* serotype 7F |  | Human blood | Denmark | KMA, Aarhus |  |
| SK677 | CCUG  55087 | *S. mitis* |  | Human mouth | Denmark | Own isolate |  |
| SK680 | 129/91 | *S. pneumoniae* serotype 19F |  |  | Australia | SSI |  |
| SK848 | 6903/91 | *S. pneumoniae* serotype 1 |  |  | Belgium | SSI |  |
| SK849 | 63/92 | *S. pneumoniae* serotype 2 |  |  | Denmark | SSI |  |
| SK850 | 785/91 | *S. pneumoniae* serotype 3 |  |  | Denmark | SSI |  |
| SK851 | 1038/91 | *S. pneumoniae* serotype 6A |  |  | Norway | SSI |  |
| SK852 | 844/91 | *S. pneumoniae* serotype 6B |  |  | Denmark | SSI |  |
| SK853 | 827/91 | *S. pneumoniae* serotype 7F |  |  | Denmark | SSI |  |
| SK854 | PK12 | *S. pneumoniae* serotype 6B |  |  | Denmark | SSI |  |
| SK856 | 911335/91 | *S. pneumoniae* serotype 14 |  |  | Denmark | SSI |  |
| SK858 | 726/91 | *S. pneumoniae* serotype 18C |  |  | Denmark | SSI |  |
| SK859 | 104/92 | *S. pneumoniae* serotype 19F |  |  | Belgium | SSI |  |
| SK862 | 6884/91 | *S. pneumoniae* serotype 23F |  |  | Denmark | SSI |  |
| SK865 | 786/91 | *S. pneumoniae* serotype 6B |  | Human blood | Denmark | SSI |  |
| SK867 | 6869/91 | *S. pneumoniae* |  |  | Belgium | SSI |  |
| SK959 | GTC849 | *S. infantis* T |  |  | Japan | Kawamura | [62]] |
| SK970 | CCUG 36756 | *S. infantis* |  | Human blood | Sweden | CCUG |  |
| SK1068 | CCUG 48465 | *S. pseudopneumoniae* |  | Human respiratory tract | U.S.A. | CCUG | [6] |
| SK1069 | CCUG 49455 | *S. pseudopneumoniae* T |  | Human sputum, pneumonia | Canada | CCUG | [6] |
| SK1121 | 2186 | *S. mitis* |  | Human blood | Denmark | SSI |  |
| SK1122 | TH1 | *S. mitis* |  |  | Japan | Own isolate |  |
| SK1123 | TOK6 | *S. mitis* |  |  | Japan | Own isolate |  |
| SK1124 | TOK3 | *S. mitis* |  |  | Japan | Own isolate |  |
| SK1125 | TOK2 | *S. mitis* |  |  | Japan | Own isolate |  |
| SK1126 | TG2 | *S. mitis* |  |  | Japan | Own isolate |  |
| SK1127 | TE1 | *S. infantis* |  |  | Japan | Own isolate |  |
| SK1128 | TI1 | *S. infantis* |  |  | Japan | Own isolate |  |
| SK1137 | TC2 | *S. mitis* |  |  | Japan | Own isolate |  |
|  | TIGR4 | *S. pneumoniae* serotype 4 |  |  |  |  | [8] |
|  | D39 | *S. pneumoniae* serotype 2 |  |  |  |  | TIGR/ J. Craig Venter Institute |
|  | G54 | *S. pneumoniae* |  |  |  |  | [Geneva Biomedical Research Institute](http://bioinfo.cnio.es/data/Spneumo/) |
|  | CGSP14 | *S. pneumoniae* |  |  |  |  | Beijing Institute of Genomics |
|  | 23F/SP-264 | *S. pneumoniae* serotype 23F |  |  |  |  | Sanger Institute |
|  | INV104B | *S. pneumoniae* serotype 1 |  |  |  |  | Sanger Institute |
|  | INV200 | *S. pneumoniae* serotype 14 |  |  |  |  | Sanger Institute |
|  | OXC141 | *S. pneumoniae* serotype 3 |  |  |  |  | Sanger Institute |

^1^ ATCC, American Type Culture Collection, Manassas, VA, U.S.A.; Brown, Tomas Brown, Gaynesville, Fl., U.S.A.; Carlsson, Jan Carlsson, University of Umeå, Umeå, Sweden; CCUG, Culture Collection of the University of Gothenburg, Gothenburg, Sweden; Cisar, John Cisar, NIH, Bethesda, MD., U.S.A.; Francioli, Patrick Francioli, University of Lausanne, Switzerland; NCTC, national Collection of Type Cultures, Colindale, London, U.K.; Sneath, Peter H. A. Sneath, University of Leicester, Leicester, U.K.; SSI, Statens Serum Institute, Copenhagen, Denmark.
