# Supplementary material for: Evolution of Streptococcus pneumoniae and Its Close Commensal Relatives
Source: PLoS One. 2008 Jul 16;3(7):e2683. doi: 10.1371/journal.pone.0002683 (PMC2444020; doi:10.1371/journal.pone.0002683)
Supplement: Table S4 — Primers used for PCR amplification and sequencing of genes. (0.03 MB DOC) [file pone.0002683.s007.doc]

**Table S4**. Primers used for PCR amplification and sequencing of genes

___________________________________________________________________________

Locus (protein) Primer sequences Purpose^1^

__________________________________________________________________________

16S rRNA 5'-TGGCTCAGGACGAACGCTGGC-3' Ampl. and seq. 5'-CGGCTGCTGGCACGTAGTTAGC-3'

*gdh*  5'-ATGGACAAACCAGCNAGYTT-3' Ampl. and seq. 5'-GCTTGAGGTCCCATRCTNCC-3'

*ddl* 5'-CTGAAAAYCAAGARGAAMTYCGTC-3' Ampl. and seq. 5'-AAAAMTTTSCTTRGCAAGRTCAACC-3'

ComCDE 5’-CATAGCTCAGCTGGATAGAGCATTCGCCTTC-3’ (2tArg2) Ampl.

5’-GGCGGTGTCTTAACCCCTTGACCAACGGACC-3’ 2tGlu

ComC 5’-CGAACGGTCGCAGGTTCGAATCCTGCTGGGATC-3’

(NPARG Arg-tRNA) Seq. Seq.

*ply* (pneumolysin) 5'-GACTTTATACTAGCTATGAATTACG-3' Hybr. probe

5'-TTTCTACCTTATCCTCTACCTGAGG-3'

*lytA* (autolysin) 5'-GTGTTATTGTAGATAGAATGCAG-3' Hybr. probe 5'-CGTGGTCTGAGTGGTTGTTTGG-3'

IS*1381* 5'-CACGATTTAAGCGTCTTGTTGGTG-3' Hybr. probe

5'-CAGCACTCAAATTCATTCGTAATCC-3'

*ply-lytA* region 5’-GGTGGTTATCCTAAGGGACGTATC-3’ Long range PCR

5’-CTCAGCCTACTAGTATAAGAAGCC-3’

*pspA* region 5’-CCTCCMCCGATRGAAATACCAGT-3’ Long range PCR

5-GACGAATCAACACCACCACTCAT-3’

*iga* region 5’-ATCGCACCAGAACAAGTYCGAGAT-3’ Long range PCR

5’-ACTACAGGACCAGCAAGWGGWCCACG-3’

*cap* region 5’-GTTKTGGCTAACTTGTCCAATG-3’ Long range PCR

5’-CTGTCAACCAAGCTTGGGC-3’

^1^Ampl., PCR amplification; Seq, sequencing; Hybr. probe, probe used for hybridization in Southern blots.
