# Supplementary figures and images for: Correction: Evolution of Streptococcus pneumoniae and Its Close Commensal Relatives
Source: PLoS One. 2009 Dec 11;4(12):10.1371/annotation/0e3332aa-1b10-4e1a-a424-546d2cb7cfff. doi: 10.1371/annotation/0e3332aa-1b10-4e1a-a424-546d2cb7cfff (PMC2795727; doi:10.1371/annotation/0e3332aa-1b10-4e1a-a424-546d2cb7cfff)

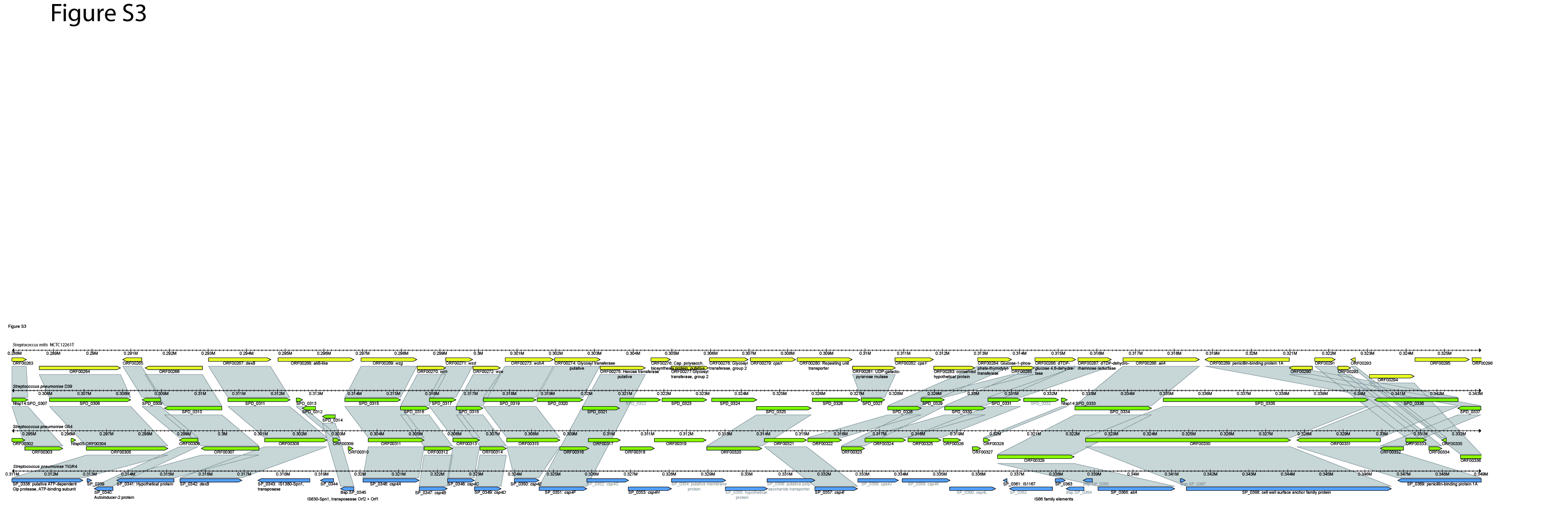

Supplement: Supplementary file 1 [file pone.0e3332aa-1b10-4e1a-a424-546d2cb7cfff.s001.tif]
